# Chitosan-based matrix as a carrier for bacteriophages

**DOI:** 10.1007/s00253-023-12838-0

**Published:** 2024-01-02

**Authors:** Monika Sikora, Sławomir Wąsik, Jacek Semaniak, Zuzanna Drulis-Kawa, Maria Wiśniewska-Wrona, Michał Arabski

**Affiliations:** 1 Department of Medical Biology, Institute of Biology, Jan Kochanowski University in Kielce, Kielce, Poland; 2 Lukasiewicz Research Network-Lodz Institute of Technology, Lodz, Poland; 3 Institute of Physics, Jan Kochanowski University in Kielce, Kielce, Poland; 4 Central Office of Measures, Warsaw, Poland; 5 Department of Pathogen Biology and Immunology, University of Wroclaw, Wroclaw, Poland

**Keywords:** Chitosan, Bacteriophages, Antibacterial dressing, Wound healing, *Pseudomonas aeruginosa*

## Abstract

**Abstract:**

Wound healing is a dynamic and complex process where infection prevention is essential. Chitosan, thanks to its bactericidal activity against gram-positive and gram-negative bacteria, as well as anti-inflammatory and hemostatic properties, is an excellent candidate to design dressings for difficult-to-heal wound treatment. The great advantage of this biopolymer is its capacity to be chemically modified, which allows for the production of various functional forms, depending on the needs and subsequent use. Moreover, chitosan can be an excellent polymer matrix for bacteriophage (phage) packing as a novel alternative/supportive antibacterial therapy approach. This study is focused on the preparation and characteristics of chitosan-based material in the form of a film with the addition of *Pseudomonas* lytic phages (KTN4, KT28, and LUZ19), which would exhibit antibacterial activity as a potential dressing that accelerates the wound healing. We investigated the method of producing a polymer based on microcrystalline chitosan (MKCh) to serve as the matrix for phage deposition. We described some important parameters such as average molar mass, swelling capacity, surface morphology, phage release profile, and antibacterial activity tested in the *Pseudomonas aeruginosa* bacterial model. The chitosan polysaccharide turned out to interact with phage particles immobilizing them within a material matrix. Nevertheless, with the high hydrophilicity and swelling features of the prepared material, the external solution of bacterial culture was absorbed and phages went in direct contact with bacteria causing their lysis in the polymer matrix.

**Key points:**

*• A novel chitosan-based matrix with the addition of active phages was prepared*

*• Phage interactions with the chitosan matrix were determined as electrostatic*

*• Phages in the matrix work through direct contact with the bacterial cells*

## Introduction

The increase in bacterial resistance to antibiotics has led to the dynamic development of research into the use of alternative therapeutics, such as phages, antimicrobial peptides, or nanoparticles. Lytic phages as natural bacterial agents are specific towards particular bactericidal strains and able to propagate where the bacteria are present thus increasing the dose at the site of infection (Linga et al. 2022). The implementation of phage therapy in the form of dressings needs a thorough analysis of phage availability, activity, and stability in the designed material. A suitable matrix, which can be semi-solid preparations, including hydrogels, creams, and ointments, as well as films or membranes, can be a carrier for the local release of phages. Due to the nature of their action, wound dressing materials can be classified into three groups: (I) passive products such as gauze and gauze-cotton composites; (II) interactive products, such as films and polymer foams ensuring transparency, water vapor permeability, oxygen permeability and additionally biodegradability; and (III) bioactive products understood as an advanced dressing capable of transporting active substances to the wound site and actively participating in the wound healing process, e.g., collagen, chitosan, or alginate (Paul and Sharma 2004; Yoon Kyung Chang et al. 2020).

The often published methods of phage immobilization applied modified polymers, such as hyaluronic acid methacrylate, hydroxypropyl methylcellulose, poly(*N*-isopropyl acrylamide), polyvinylpyrrolidone, cellulose diacetate, or gelled milk proteins. The phages were immobilized in various structures, in terms of composition and morphology (Malik et al. 2017). Phages were also encapsulated in nanospheres, microspheres, nanofibers, microfibers, membranes, and thin-layer biostructures. Encapsulation of phage, e.g., in alginates, takes place under mild processing conditions, in which the alginate is gelled by cross-linking between carboxylate anions of guluronic acid and calcium ions (Ergin et al. 2020). One of the disadvantages of oral administration of phages resulting in the loss of infective phage particles is the exposure to extreme environmental conditions such as high acidity in the stomach and the presence of enzymes operating in the digestive system (Veverka et al. 2020). After systemic administration, phages may be cleared or inactivated by the host’s immune system or activated with a delay (Hosseinidoust et al. 2014). Another problem often described for topical application is the difficulty in improving the stability of phages in the polymer matrix. One of the proposed solutions is phage product preparations in the form of a dry powder. Powders for pulmonary administration and semi-liquid hydrogel formulations for topical application to the skin are good examples. Powders have an advantage over liquid preparations due to their ease of storage, transport, and administration (Silva et al. 2021). The factors initiating the release of phages from the formulation matrix are mainly temperature, change in pH, or external enzymes. Polymer matrices are subjected to various changes and modifications such as degradation, polymer solvation, dissolution, hydrolysis, phase inversion, or swelling. A big challenge is the proper stabilization of the matrix templates and controlling the release of phages into the environment (Batalha et al. 2021). There are few reports describing the form of films as a carrier for phages dedicated to the treatment of superficial skin wounds. The solution proposed in the film system eliminates the problem of controlling the kinetics of phage release into the wound environment because the phages interact with bacteria directly in the polymer matrix. Thanks to the high hydrophilicity of the film membrane used, bacteria from the wound environment were absorbed into the polymer matrix. The phages maintained as infective within the matrix were able to effectively propagate in bacterial cells present in the polymer matrix. The microcrystalline chitosan may be such example of a polymer matrix carrier used for phage immobilization (Yan et al. 2021).

Chitin and chitosan are naturally occurring polymers with versatile applications in many fields. Chitin is usually obtained from natural sources, such as the outer shells of marine crustaceans, outer skeletons of insects, and fungal cell walls. Chitosan is a polysaccharide composed of a deacetylated part (β-[1,4]-D-glucosamine) and an acetylated part (*N*-acetyl-D-glucosamine), obtained by partial deacetylation of chitin (Rochima et al. 2017; Wu et al. 2005). It is a non-toxic, biocompatible, and biodegradable polymer. It is also easily subject to any chemical and enzymatic modification; therefore, it makes it possible to use it in various forms, e.g., in the form of a gel, sponge, film, fiber, or granulate. These unique properties enable its application in medicine, pharmacy, and tissue engineering as well as in various industries. Chitosan can bind negatively charged micromolecules and macromolecules to positively charged functional groups in its chain, providing hemostatic and antibacterial properties important for medical use (Kedzierska and Milowska 2019). Chitosan as a dressing formulation proves that the form of the membrane also can stimulate the migration of inflammatory cells during wound healing (Biagini et al. 1992; Minami 1993). Therefore, it is a good candidate for the design of new dressing matrices that are a carrier of active compounds with antibacterial properties. Moreover, with flexibility and a thin membrane structure, it adheres perfectly to the freshly wounded skin surface, reduces itching and pain, and accelerates the scarring process thanks to improved epithelization and collagen deposition (Azad et al. 2004; Howling et al. 2001; Mi et al. 2003). When designed to be combined with another antibacterial agent such as phages, it is important to carefully adjust all parameters, both the physicochemical properties of the polymer matrix itself, as well as the biological features of phages to achieve efficient activity at the site of application (Bourdin et al. 2014; Koskella 2014).

In this study, we prepare chitosan-based dressing material in the form of a film with the addition of active phages, which would exhibit antibacterial activity under conditions that accelerate the wound-healing process. Thanks to the high hydrophilicity of the membrane used, phages may act in contact with bacteria directly in the polymer matrix. We describe a method of producing a polymer matrix based on microcrystalline chitosan (MKCh) and selected the best composition of the polymer matrix for phage immobilization. Three lytic *Pseudomonas aeruginosa* phages (KT28, KTN4, and LUZ19) differing in taxonomy classification, capsid size, and morphology, as well as recognized receptors, have been selected for biocomposite preparation to evaluate the release profiles as well as potential interference with chitosan matrix. Finally, the antibacterial properties of the prepared biocomposite were tested against *P. aeruginosa* PAO1, as representative of the most common pathogen causing wound infections.

## Materials and methods

### Bacterial strain and phages

The *P. aeruginosa* PAO1 (ATCC 15692) strain purchased from the American Type Culture Collection (ATCC, Manassas, VA, USA) was used as a bacterial model. Three lytic phages specific to *P. aeruginosa* PAO1 differing in taxonomy classification, receptor specificity, and virion size were used as model phages. KTN4 giant *Phikzvirus* and KT28 Myovirus *Pbunavirus* originated from the collection of the Department of Pathogen Biology and Immunology, University of Wroclaw, Wroclaw, Poland (Danis-Wlodarczyk et al. 2015, 2016), whereas LUZ19 Podovirus was kindly provided by Rob Lavigne from the Laboratory of Gene Technology, KU Leuven, Leuven, Belgium (Ceyssens et al. 2010). The colony count of bacteria (CFU/mL; colony-forming unit) was assessed using trypticase soy agar (TSA, Becton Dickinson and Company, Cockeysville, MD, USA), and bacteria were stored at − 70 °C in trypticase soy broth (TSB, Becton Dickinson and Company, Cockeysville, MD, USA) supplemented with 20% glycerol. The phage titre (PFU/mL; phage forming unit) was determined using the standard spot technique on TSA, and phage filtrates in TSB were stored at 4 °C. Phage characteristics are presented in Table 1.

**Table 1:**
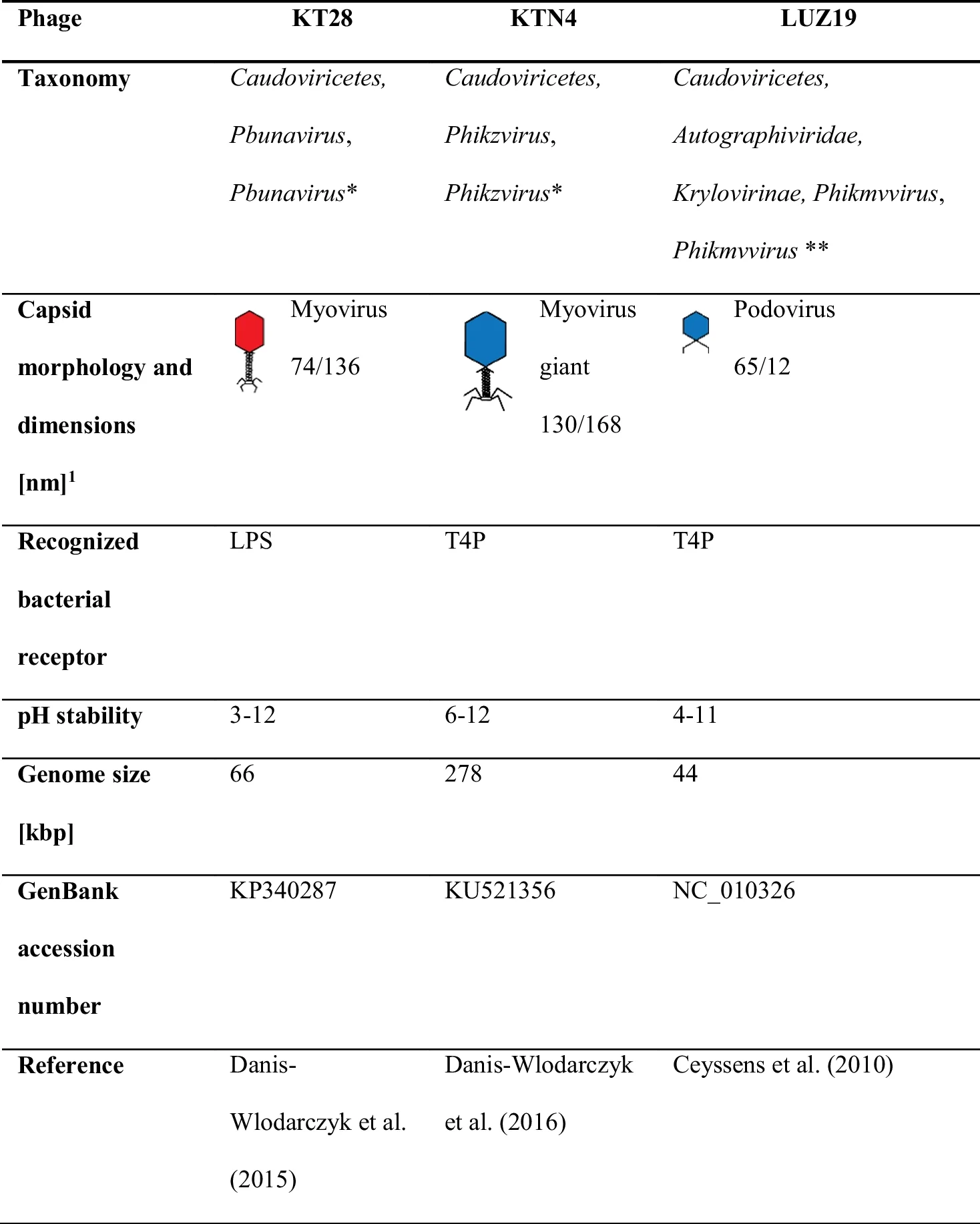
Characteristics of phages selected for the study

^1^Approximated dimensions of head diameter/tail length

*Collection of the Department of Pathogen Biology and Immunology, University of Wroclaw, Wroclaw, Poland

**Collection of the Laboratory of Gene Technology, KU Leuven, Leuven, Belgium

### Synthesis of the polymer matrix

**Fig. 1:**
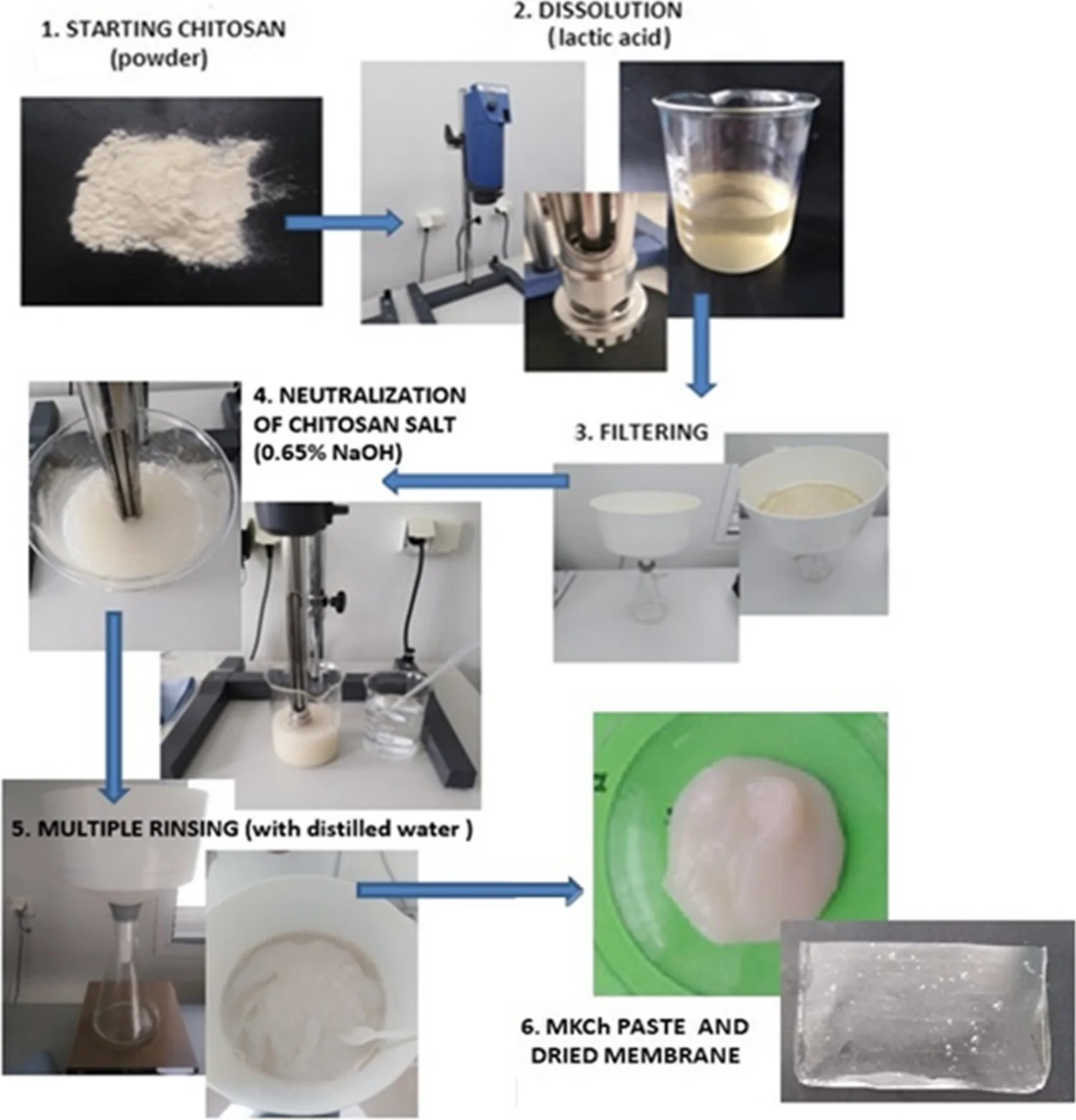
Scheme of preparation of MKCh membranes from chitosan

Medical-grade shrimp chitosan ChitoClear fg 95 (TM 4293) from Primex ehf Iceland Company (Siglufjordur, Iceland) was used to produce the polymer base. The basic physical and chemical parameters of the starting raw material are performed and described in Table 2. Microcrystalline chitosan (MKCh) was produced by agglomeration from a solution, by a continuous method developed at the Lukasiewicz Research Network-Lodz Institute of Technology according to patent (Struszczyk et al. 1991) (Fig. 1).

**Table 2:** Basic physicochemical properties of chitosan ChitoClear fg 95 (TM 4293)

*M*_*v*_^1^ (kDa)	DD^2^ (%)	Ash content^3^ (%)	Heavy metal content^4^ (%)	SSI^5^ (%)
235.0	86.8	0.60	As < 0.2; Cd < 0.2; Pb < 1.0; Zn = 0.23; Hg < 0.01	224.59

^1^Average molar mass determined by the viscometric method, kDa

^2^Degree of deacetylation determined by the spectrophotometric method, consisting in determining the maximum of the curve of the first derivative of the UV spectrum

^3^Ash content was determined by weight, based on the ash residue of the sample at 800 °C

^4^The content of heavy metals determined with the use of atomic absorption spectrometry

^5^Secondary swelling index (SSI) was determined gravimetrically

In the first stage of the process, a starting chitosan solution was prepared in an aqueous diluted lactic acid solution (Avantor Performance Materials, Gliwice, Poland) with a concentration of 0.45 wt% and chitosan (Primex ehf., Siglufjordur, Iceland) concentration of 1% by weight. In this stage of the process, an IKA WERKE T50 basic homogenizer (Staufen im Breisgau, Germany) was used with an ending that allows for grinding the preparation in the form of a suspension in the range of 10–100 µm (G45G)—the process was carried out at a speed of 4800–6400 rpm for 15 min. The polymer salt solution was then mixed with a stirrer at a reduced speed of approx. 4000–5200 rpm for 4–6 h (intermittently to avoid overheating of the polymer solution). To stabilize the pH value and deaeration, the obtained chitosan lactate solution was left overnight, and storing it in a refrigerator. After 24 h, the solution was filtered on a Büchner funnel equipped with a BT 192 batiste cloth (Witan, Warsaw, Poland). The chitosan salt solution was then neutralized with diluted sodium hydroxide (Chempur, Gliwice, Poland) with a concentration of 0.65% by weight until the pH of the obtained suspension is in the range of 7.4–7.6 (20 °C). The MKCh dispersion was purified by repeated washing with distilled water until the anion reaction of the removed salt ceased (until the pH dropped to approx. 7.2–7.4; 20 °C). Washing was carried out using the membrane technique in the ultrafiltration process or using a nipper equipped with a filter material composed of batiste and non-woven fabric PET-PP (Witan, Warsaw, Poland). Free of salt and excess alkali, the MKCh suspension was concentrated to a paste consistency, which was the basic formulation.

The produced microcrystalline chitosan was used to prepare a paste dilution to 1.5% with microbiologically pure water with the addition of glycerin (Sigma-Aldrich, St. Louis, MO, USA) (glycerin was added in the amount of 1:0.6 with the polymer content). Glycerin in the presented system acts as a plasticizer that gives the chitosan membrane flexibility. The diluted MKCh paste prepared in this way in the amount of 5 g was poured on Teflon plates with window sizes 5 × 6 cm and mixed with 1 mL of phage (10^8^ PFU/mL; phage forming unit). In this proportion, 4 membranes were made for each matrix (1, 2, and 3). Phages were dissolved in TSB medium, which was also added to the control probe. It was left to dry at room temperature in sterile conditions. The obtained polymer matrix with a single phage was used in this study.

### Physicochemical parameters of microcrystalline chitosan (MKCh)

The MKCh used for matrix 1, 2, or 3 syntheses was characterized by the viscometric method for the determination of the average molar mass. Moreover, the secondary swelling index of MKCh and chitosan membranes was determined. The viscometric average molecular weight was calculated from the intrinsic viscosity [*η]* using the Mark-Houwink formula (according to the system of internal procedures: SPR/BPB/5—Determination of the viscosimetric average molecular weight, according to GLP Nr. G-016; Lukasiewicz, Lodz Institute of Technology, Poland):

1 $$\left[{{\eta}}\right]={{k}}\times \overline{{{{M}} }_{{{V}}}^{{{a}}}}$$

where,

[*η*] is limit viscosity number, K; *α* is empirically determined constants amounting to *k* = 8.93 × ${10}^{-4}$; *α* = 0.71; $\overline{{\mathrm{M} }_{\mathrm{V}}}$ is viscometric average molecular weight. Viscosity measurements were made on a dilution viscometer with a capillary no. 1, *K* ≈ 0.01.

The secondary swelling index (SSI) of MKCh and polymer matrices with MKCh was determined gravimetrically based on the equation (according to the system of internal procedures: SPR/BPB/13—Determination of moisture content in chitosan, according to GLP Nr. G-016 Lukasiewicz, Lodz Institute of Technology, Poland):

2 $${{S}}{{S}}{{I}}=\left[\left({{{m}}}_{1}-{{{m}}}_{0}\right){:{{{m}}}_{0}}\right]\times 100\boldsymbol{\%}$$

where,

*m*_1_ is the mass of the MKCh sample/polymer matrix after being kept in water for 20 h and centrifugation (4000 rpm) for 10 min and *m*_0_ is the mass of the MKCh sample/polymer matrix after drying at 105 °C to a stable weight.

### Scanning electron microscopy

The surface structure of matrix 3 modified by phages was assessed using a Quanta 200 Scanning Electron Microscope (SEM) (FEI Co., Alhambra, CA, USA). The samples were mounted on a jaw table and then sprinkled with a thin layer of gold (on the order of 20 nm) using a Q 150R S vacuum sprayer (Quorum Technologies, Ltd, Lewes, UK). After spraying, the samples were placed in the microscope chamber. The tests were carried out in high vacuum conditions with a voltage accelerating the 5 kV electron beam, using the analySISDocu program by Soft Imaging System, adapted to work in the Quanty environment (Soft Imaging System, Muenster, Germany).

### Laser interferometry method

The laser interferometry method was used to quantify the number of released phages and non-polymerized matrix components (NMCs) from the polymer product in the function of time. The laser interferometry system consists of a two-beam Mach–Zehnder interferometer with a He–Ne laser type HN 40P (Zeiss, Oberkochen, Germany), a cuvette (internal dimensions 70 mm high, 10 mm wide, optical path length 7 mm) made with optical glass of high uniformity, a TV-CCD camera (charge-coupled device television camera), and a computer with software for the acquisition and processing of interference images (interferograms) (Arabski et al. 2007; Dworecki et al. 2006; Rewak-Soroczynska et al. 2021). The phage-modified matrix 3 was placed at the bottom of the cuvette which was then filled with water. The transport properties of phages/NMC from the polymer matrix to water were measured using the laser interferometry system based on obtained interferograms. A computer image-processing system, complete with dedicated software, enables mathematical analysis of interferograms shown on the system screen. Moreover, taking a series of pictures of interference images in time and conducting a mathematical analysis thereof enables quantitative analysis of real-time release kinetic phages/NMC in near-polymer surface fields. The interferograms, which appear due to the interference of laser beams, were determined by the refraction coefficient of the solute which in turn depends on substance concentration. When the solute is uniform, the interference fringes are straight, and they bend when a concentration gradient appears. So, the laser interferometry method was also used in the studies of phages/NMC released from polymer at 37 °C for 2 h. For this purpose, the total amount of phages/NMC released was analyzed. Since the proportionality constant between the concentration and the refractive index for the phages/NMC released in this experiment is unknown, the sums of the changes in the refraction index (SCRI) of the solute were calculated:

3 $${\mathrm{SCRI}}\left(t\right)=S\underset{0}{\overset{\delta }{\int }}\Delta n\left(x,t\right)dx.$$

The SCRI(*t*) reflects the time dependence of the total amount of phages/NMC (in arbitrary units) released from the polymer to the water phase. Interferograms were recorded in the time interval Δ*t* = 5 min from 5 to 120 min at the temperature of 37 °C. Moreover, using a laser interferometry system, the thickness of the modified matrix 3 (its swelling) was measured during the release of the phages/NMCs to the water environment. To this aim using the software dedicated to the analysis of interferograms, the coordinates of the area of a given membrane in the interference image were determined. Based on the obtained coordinates, the thickness of the modified matrix 3 in pixels was determined. Then, knowing the pixel size, the membrane thickness in mm was calculated. For the optical magnification used in our measuring system during the experiment, the pixel size was equal to 0.019 mm.

### Activity of phages released from a matrix 3

Squares with dimensions of 2 cm × 2 cm (native and phage modified) were cut out of the prepared matrix 3 and used to analyze by two microbiological culture methods. For experiments, bacterial culture was refreshed in TSB and incubated at 37 °C for 18 h.

The first method was to assess whether phages eradicating *P. aeruginosa* PAO1 bacteria are released from the matrix 3. For this purpose, polymer matrices in the form of cut squares of 2 cm × 2 cm (native and phage-modified) were flooded with 2 mL of microbiologically pure water and left at 25 °C for 18 h. After the incubation time, 90 µL of post-incubation water was taken from each test sample and placed in a sterile 90-well microtiter plate. To each well of post-incubation water, 100 µL of broth (TSB tryptone soy broth, Thermo Scientific, Waltham, MA, USA) and 10 µL of bacterial culture containing 10^5^ *P. aeruginosa* PAO1 cells were added. The bacterial growth kinetics were measured spectrophotometrically at 600 nm for 18 h at 37 °C with a 0.5-h time interval using a TECAN SPARK spectrofluorometer (Tecan Group Ltd., Männedorf, Switzerland) (Ergin et al. 2020).

The second method was to determine whether prepared matrix 3 absorb bacteria during their swelling, and then were eliminated by the phages present in their structure. The above antimicrobial properties of the polymer matrix were evaluated on the *P. aeruginosa* PAO1 strain using the modified ISO 22196:2007(E) (https://www.iso.org/standard/40759.html). For this purpose, 200 µL of a bacterial culture containing 10^5^ cells of *P. aeruginosa* PAO1 was placed on matrix 3 with dimensions of 2 cm × 2 cm (native and modified with phages), and the reduction level was determined according to ISO 22196:2007 (2011).

### Fourier-transform infrared spectroscopy

The analysis of matrix 3 structure and their interactions with phages, based on the identification of functional groups, was performed using the Fourier-transform infrared spectroscopy (FT-IR), according to the system of internal procedures: SPR/BLF/14-FT-IR infrared spectrometry, according to GLP No. G-016, Lukasiewicz, Lodz Institute of Technology, Poland. FT-IR spectra were recorded on a Genesis Series FT-IR instrument equipped with the ATI Mattson and Peak Solve analytical software (Unicam, Dowlish Ford, UK). The spectra were made using the X-ray technique in the wavenumber range of 500–4000 ${\mathrm{cm}}^{-1}$, with a resolution of 4 ${\mathrm{cm}}^{-1}$, and the number of scans was 32. Preparations in the form of KBr tablets were prepared using solid weights. The polymer sample was mechanically ground, and 1 mg was taken, next pre-mixed with dried KBr 100 mg, and the first tablet using a press at a pressure of 70 atm. for 5 min with a vacuum was made. The tablet was ground in a lab ball mill to thoroughly disperse the polymer in the carrier. The sample was dried for 1 h in a laboratory dryer at 70 °C to remove moisture while preparing tablet. The sample was then compressed at 70 atm for 5 min with vacuum and transferred to the FT-IR transmission chamber. Background measurement was performed before sample measurement. In transmission techniques, the oscillation spectrum is analyzed by measuring the intensity of the radiation after it has passed through the sample. The decrease in the intensity of the incident beam indicates the absorption of the radiation by the sample. In transmission techniques, a measure of the absorption of radiation with a specific wavenumber (*ṽ*) through the sample can be either transmittance (*T(ṽ)*) or absorbance (*A(ṽ)*), which are defined by Eqs. (4) and (5). Absorbance is a practical value because it can be used to quantify absorption—its size is directly proportional to the amount of absorbing particles according to the Bouguer-Lambert-Beer law.

4 $${{T}}\left(\widetilde{{{\nu}}}\right)=\frac{{{I}}}{{{{I}}}_{0}}$$

5 $${{A}}\left(\widetilde{{{\nu}}}\right)={{l}}{{o}}{{g}}\frac{{{{I}}}_{0}}{{{I}}}=-{{l}}{{o}}{{g}}{{T}}$$

where,

*I*_0_ is the intensity of the beam incident on the sample and *I* is the beam intensity after passing through the sample.

## Results

**Fig. 2:**
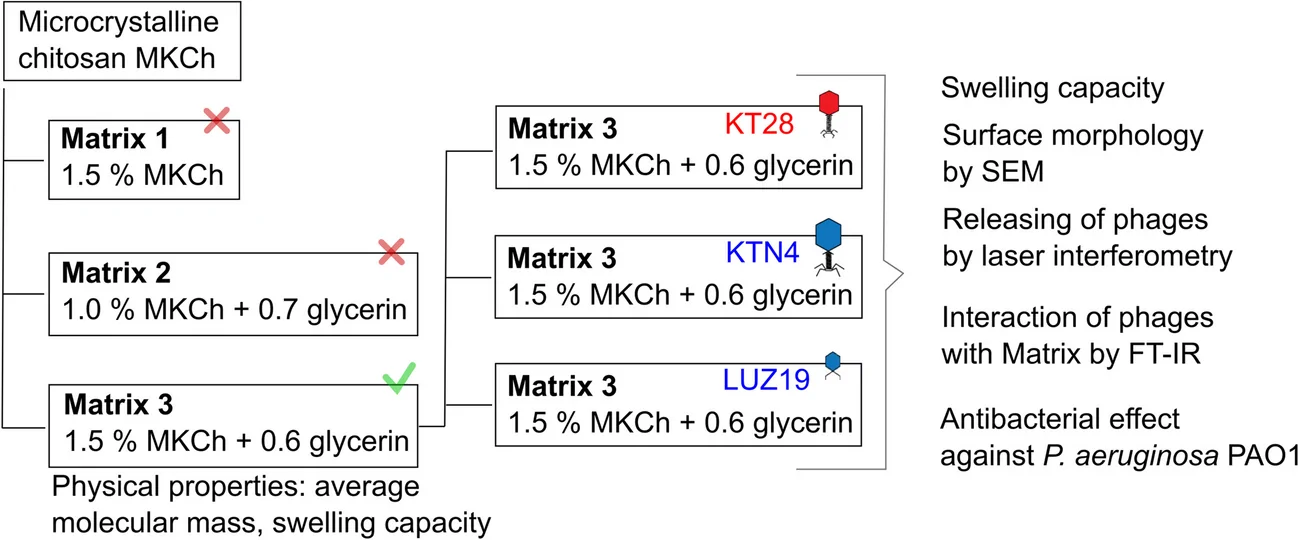
The scheme of the experimental pipeline of the study

The first step was to select the appropriate composition of the polymer matrix, which was then to be combined with three different lytic phages (KT28, KTN4, and LUZ19) (Fig. 2).

Three matrices in the form of a film (matrix 1, matrix 2, and matrix 3) were prepared from microcrystalline chitosan MKCh. MKCh chitosan was previously produced by agglomeration from a solution of 1% chitosan in 0.45% lactic acid (description of the method, Fig. 1). It seems to be important that chitosan in MKCh form has hydrophilic properties, and it is crucial for the design of antibacterial matrix based on phages activity. The produced matrixes differed in composition: matrix 1 contains 1.5% MKCh, matrix 2 contains 1.0% MKCh with glycerin (in a ratio of 1:0.7 to the polymer content in the produced microcrystalline chitosan), and matrix 3 contains 1.5% MKCh with glycerin (in a ratio of 1:0.6 to the polymer content in the produced microcrystalline chitosan). When choosing the right matrix, the main considerations were the appropriate mechanical properties, flexibility, and tear resistance, while maintaining the highest possible concentration of chitosan. Matrix 3 was chosen for its best flexibility properties. Matrix 3 showed the best flexibility while maintaining the correct ratio of glycerin to the polymer. The higher percentage of chitosan (1.5% MKCh) in matrix 3 increases the potential biological properties of the films than matrix 2 (1.0%). Matrix 1 without the addition of glycerin crumbled and did not show plasticity. Therefore, matrix 3 was chosen to be modified by phages in this study.

Matrix 3 selected for further research was checked in terms of the loss of molecular mass after the synthesis procedure. The agglomeration of MKCh might be correlated with the reduction of the molecular mass of chitosan; thus, the average molar mass of matrix 3 alone was determined. Matrix 3 was then combined with selected *P. aeruginosa* phages to determine the differences in viral particle release efficiency conditioned by the virion size and affinity to polysaccharide or protein receptors. Three phages were selected: KT28 Myovirus with a middle capsid size (74/136 nm) specific to lipopolysaccharide (LPS), KTN4 Myovirus giant (130/168 nm), and small LUZ19 Podovirus (65/12 nm) adsorbing to type IV pili (T4P) (Table 1). Prepared phage containing biocomposite (matrix 3) was tested for several features: swelling capacity, surface morphology by SEM, ability to release phages tested by laser interferometry system as well as the interactions of phages with chitosan matrix measured by FT-IR. The biocomposite in the form of a film was in the end evaluated for the antibacterial effect against *P. aeruginosa* PAO1 strain.

### Physicochemical properties of matrix 3 as hydrophilic absorption material

Firstly, the average molar mass of matrix 3 (1.5% MKCh+ glycerin in a ratio of 1:0.6 to the polymer content in the produced microcrystalline chitosan) was determined by viscometry. The MKCh sample for this measurement was prepared in the form of a film (paste dried at room temperature) and dissolved in 25 mL of solvent (0.2 M acetic acid + 0.1 M sodium chloride + 4M urea). The determination of the weight takes into account the moisture content of the sample (%). Viscosity measurements were made on a dilution viscometer with a capillary no. 1, *K* ≈ 0.01 (Table 3).

**Table 3:** Physical parameters of matrix 3 measured by the viscometric method for chitosan molecular mass determination

Sample mass (g)	Volume^1^ (mL)	*T*_0_^2^ (s)	Moisture^3^ (%)	*k*^4^	*α*^4^	[*µ*]^5^	*M*_*v*_^6^ (kDa)
0.0393	25	111.39	4.85	8.93 × 10^−4^	0.71	5.641	225.3

^1^Volume of used solvent

^2^Solvent drain time

^3^Moisture content of the test sample

^4^*k* and *α* are constant values determined empirically

^5^Limit viscosity number

^6^Average molar mass

The molecular mass of chitosan in matrix 3 (225.3 kDa) turned out to be lower in comparison to powder chitosan (235 kDa) used for matrix synthesis, but the level of molecular mass reduction was low indicating that chitosan was not degraded during matrix 3 preparation. The difference in molecular mass of chitosan might be associated with the reduction of water molecules in the matrix structure.

Nextly, the secondary swelling index (SSI index %) of the synthesized polymer matrix was measured to determine the ability to absorb water, which has great importance in the context of creating absorbent dressings. The MKCh paste dissolved to 1.5% solution using water was formed into matrix 3 combined with each phage alone (KTN4, KT28, or LUZ19). Matrix 3 was poured on Teflon plates, left to dry at room temperature, and characterized in terms of swelling capacity (SSI index %). The high swelling index of tested membrane biocomposite proved the high water absorption capacity also for the film-containing phages (Table 4).

**Table 4:** Swelling capacity (SSI index %) and thickness of matrix 3 alone and modified with phages. The matrix thickness was measured by laser interferometry after 5 min and 120 min after water adding (*n* = 3)

Polymer	Water-matrix 3 interaction parameters		
	SSI index % (%)	Laser interferometry [thickness (mm)]	
		5 min	120 min
Matrix 3	366.67	0.728 ± 0.055	0.785 ± 0.011
Matrix 3 + KTN4 Myovirus giant	390.32	0.342 ± 0.027	0.345 ±0.097
Matrix 3 + KT28 Myovirus	195.16	0.903 ± 0.013	0.884 ± 0.148
Matrix 3 + LUZ19 Podovirus	256.41	0.504 ± 0.067	0.495 ± 0.027

Matrix 3 alone and modified with phages had 2–4 times lower swelling capacity than MKCh paste form (714.00%; SSI index). It is related to the decrease of chitosan concentration in tested polymers in comparison to the chitosan-concentrated paste form. The highest swelling capacity was found for matrix 3 with KTN4 giant, nextly with LUZ19 Podovirus, and the lowest for one combined with KT28 Myovirus. The virion size of phages was not correlated with the swelling properties of matrix 3. Additionally, the laser interferometry method was used to verify the binding of water with matrix 3 by measurement of the thickness of swollen matrix 3 compared to the dried form with ~ 0.274-mm thickness. The highest thickness of matrix 3 modified with KT28 was observed, nextly for LUZ 19 and the lowest for KTN4.

Generally, KT28 phage-recognizing sugars (LPS) had the highest chemical ability to bind with chitosan-based matrix 3. It seems to correspond to the lower level of the SSI index. The laser interferometry showed that matrix 3 had hydrophilic properties, and the maximum water-binding properties of all tested polymers were observed after 5 min and statistically no-changing after 120 min. of the experiment. This might be correlated with the hydrophilic properties of bounded phage virions and/or the interaction of water molecules with other chemical groups of chitosan. Both tests confirm the ability to swell and absorb liquids.

**Fig. 3:**
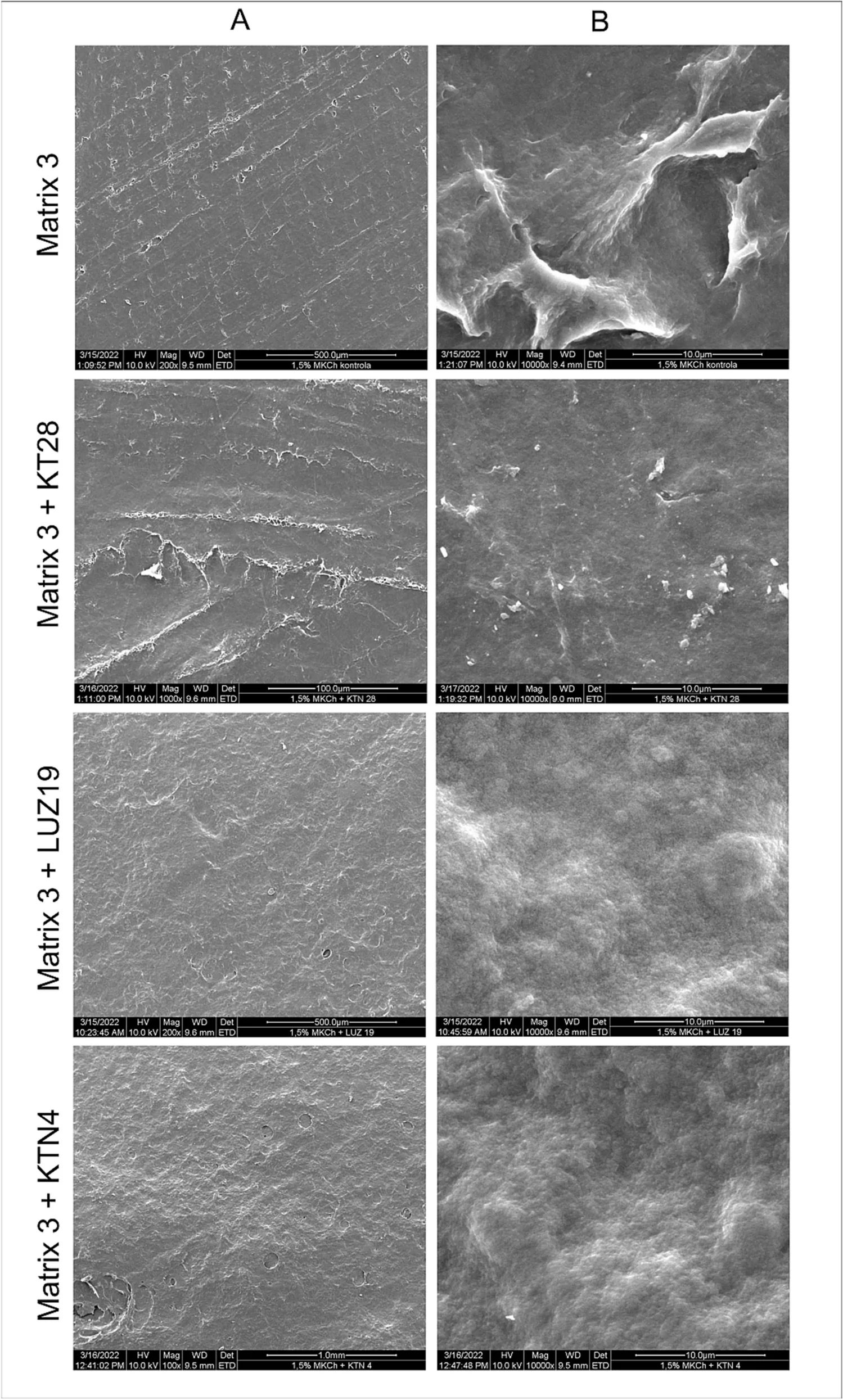
Photographic documentation of matrix 3 alone and modified with phages observed by SEM. Column A, magnification 100× and column B, magnification 10,000×

Finally, the surface morphology of matrix 3 alone and modified with phages was assessed using SEM (Fig. 3). The surface morphology was important as an adherent one to the wound site. The surface of matrix 3 alone and as phage-modified forms were similar, which confirmed no negative effect of phage addition on the polymer morphology.

### Releasing of phages from swelling chitosan-based matrix 3

**Fig. 4:**
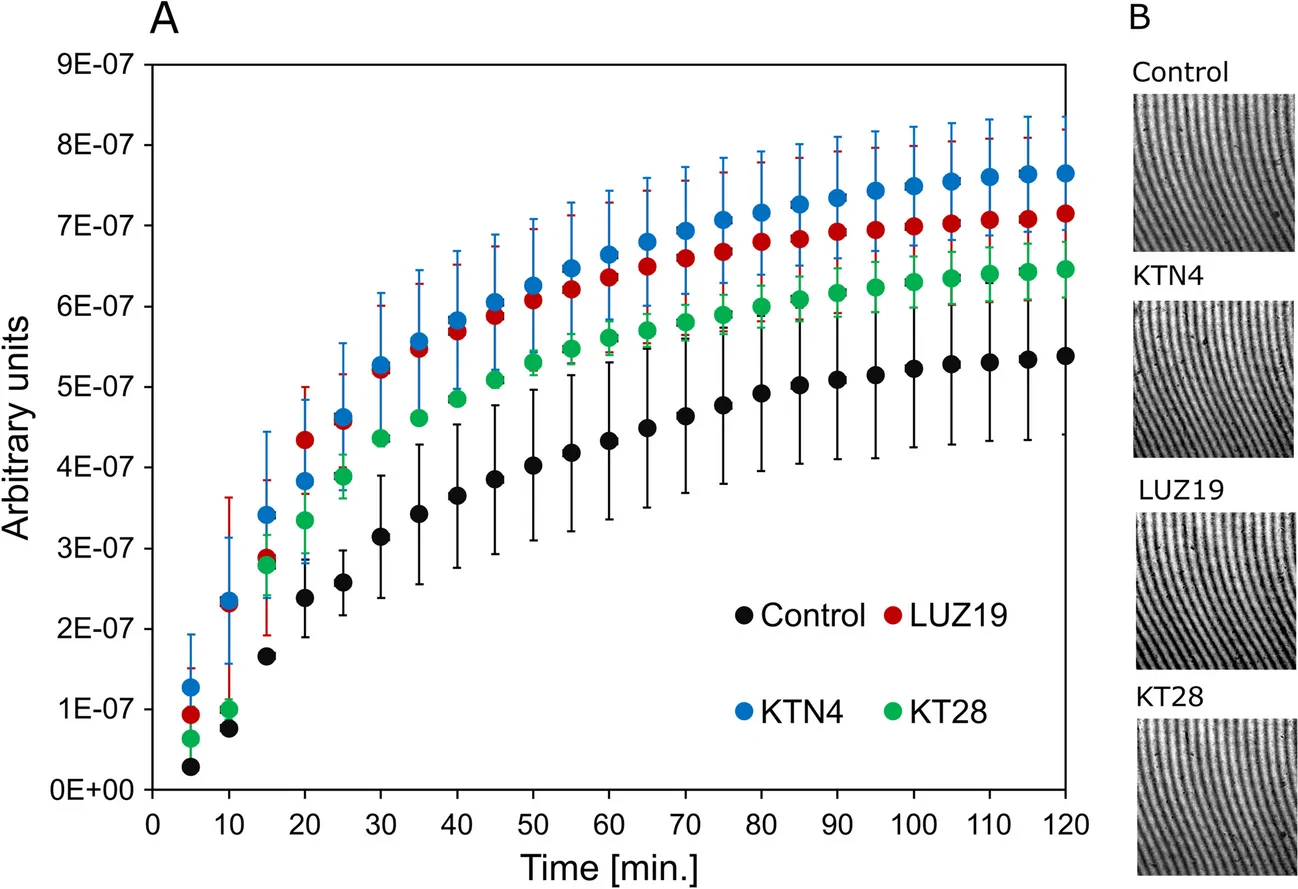
**A** The sums of the changes in the refraction index (SCRI) of the solution (a.u.) for phages and non-polymerized matrix components (NMCs) released from matrix 3 at 37 °C. **B** The examples of interferograms at 120-min time point; control—matrix 3

The laser interferometry method was used to analyze the release efficiency of phage virions from the swelling matrix 3. Figure 4 shows the phages and NMCs releasing from matrix 3 in the function of time at 37 °C. The SCRI of the solution during the release of phages and NMC is presented in arbitrary units. These sums correspond directly to the number of phages and/or NMCr released from the proper matrix 3. All phages were released similarly to each other regardless of their size and virion morphology. Moreover, the release profiles compared to the control showed no statistically significant differences which means phages were immobilized within matrix 3 structure.

### Interaction of phages with matrix 3 components measured by FT-IR

**Fig. 5:**
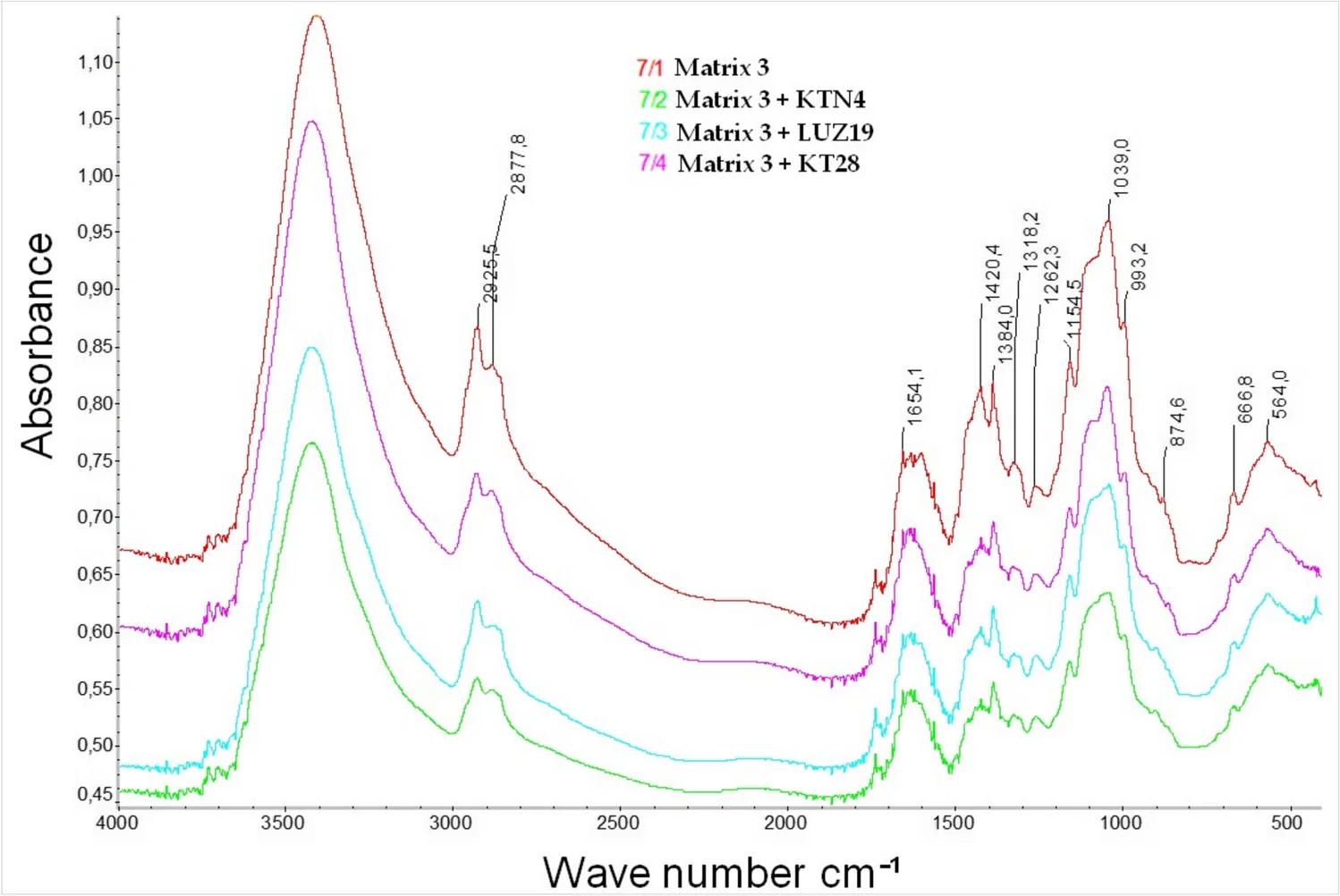
FT-IR spectra for matrix 3 alone (red) compared to matrix 3 modified with each phage: LUZ19 (cyan), KTN4 (green), and KT28 (magenta)

The interferometric study of phage-modified matrix 3 showed the limited release of phages from this polymer, which suggested a possible binding of phage particles to the matrix macromolecules. Therefore, an FT-IR study was carried out to check the presence of bonds that might have been formed between the matrix and phage particles. The analysis of the matrix 3 interactions with each phage (LUZ19, KTN4, and KT28) was based on the identification of functional chitosan groups. Figure 5 shows FT-IR spectra for matrix 3 without phages in comparison with the matrix containing selected phages.

We observed a strong peak around 3490 ${\mathrm{cm}}^{-1}$ corresponding to O–H stretching, and peak around 2877 ${\mathrm{cm}}^{-1}$ attributed to C–H asymmetric stretching. These peaks are characteristic of the polysaccharide nature of chitosan. The presence of *N*-acetyl groups was confirmed by the two peaks around 1654 ${\mathrm{cm}}^{-1}$ for C=O stretching of amide I and 1318 ${\mathrm{cm}}^{-1}$ for C–N stretching of amide III. The absorption peak at 1154 ${\mathrm{cm}}^{-1}$ attributed to asymmetric stretching of the C–O–C bridge. All above-described peaks occurred both in pure chitosan (matrix 3) and for matrix 3 combined with phages, but no differences were observed.

As a result of the FT-IR analysis using the transmission technique, a flattening of the peak characteristic of the bonds in the saccharide ring (1039 ${\mathrm{cm}}^{-1}$) of chitosan was observed for biocomposite with LUZ19 and KTN4 phages, and absent for matrix 3 modified with KT28 Myovirus. In the chitosan, this peak was distinct, which may indicate the binding of phages LUZ19 and KTN4 to the structure of the saccharide rings in the polymer matrix. The decrease in the intensity of the peaks observed for all tested phages’ addition suggested also a slight degree of polymer degradation. Flattened two peaks were observed around 1420 ${\mathrm{cm}}^{-1}$ (symmetric deformations of CH_2_) and 2925 ${\mathrm{cm}}^{-1}$(C–H symmetric stretching) for all phage-containing matrices. When comparing the peak of 1420 ${\mathrm{cm}}^{-1 }$ with the adjacent peak 1384 ${\mathrm{cm}}^{-1}$ and 2925 ${\mathrm{cm}}^{-1}$ with 2977 ${\mathrm{cm}}^{-1}$, the change in intensity was significant. The reduction in the intensity of these peaks may be due to the use of these groups for binding phages. These are easily accessible groups that can form hydrogen bonds.

Taking into account the FT-IR analysis, two possibilities for phage interactions were proposed. The first mechanism is characteristic of phages LUZ19 and KTN4 which could bind with saccharide rings of chitosan in matrix 3 structure. The second is regarding KT28 Myovirus, which might interact with CH_3_ groups by hydrogen bonds.

### Antibacterial effect of matrix 3 modified by lytic phages

The antibacterial activity of the matrix 3 containing lytic phages as potential dressing material was tested against the *P. areuginosa* PAO1 strain. Two methods of analysis were used to assess whether the tested designed dressings exhibit antibacterial activity through the active release or static activity of the phages embedded/immobilized in the matrix.

The first method consisted in assessing the phages released from matrix 3 to 2 mL of microbiologically pure water followed by the eradication capacity of *P. aeruginosa* PAO1. To determine the susceptibility to phage-mediated lysis, the growth kinetics of *P. areuginosa* PAO1 was measured in the presence of released phages in the water solution. The absorbance at 600 nm was measured for 18 h at 37 °C, and the colony-forming unit per mL (CFU/mL) of *P. aeruginosa* PAO1 was determined for the native matrix (control) and modified with phages (KTN4, KT28, and LUZ19). There were no statistically significant differences in the absorbance value for control (0.34 ± 0.01) and phage-modified matrix 3: KTN4 (0.38 ± 0.02), KT28 (0.39 ± 0.02), and LUZ19 (0.34 ± 0.01). The lack of these differences indicates that the phages suspended in the polymer matrix are not released from its structure. The lack of significant diffusion of phage particles within the chitosan matrix might be explained by the difference in net charge between the capsid (negatively charged) and the tail (positively charged) causing directional immobility in the positively charged biopolymer matrix.

The second method determined the capability of polymer matrices to absorb bacterial cells during swelling and then eliminate them by the lytic activity of phages immobilized in the polymer structure. Two hundred microliters of *P. aeruginosa* PAO1 (2.37 ± 0.86 × 10^6^ CFU/mL) was incubated with the native matrix 3 (no phage addition) and matrix 3 modified with phages (KTN4, KT28, and LUZ19) in a water bath for 24 h at 37 °C. The colony-forming unit (CFU/mL) was determined at time 0 (*T*_0_) and after 24 h of incubation (*T*_24_) using the plate method. The CFU/mL of *P. aeruginosa* PAO1 cells after 24 h in contact with tested matrix 3 alone or modified with phages in comparison to the initial 2.37 ± 0.86 × 10^6^ CFU/mL was determined. The obtained results (CFU/mL) after 24 h of incubation were as follows: for the control (PAO1) 4.96 ± 2.58 × 10^6^ and for matrix 3 2.32 ± 1.46 × 10^6^. For matrix 3 with KTN4 Myovirus giant, KT28 Myovirus as well as matrix 3 with LUZ19 Podovirus no growth of PAO1 colonies was observed after 24-h incubation.

Matrix 3 alone was found not to have a strong antibacterial effect in contrast to the phage-containing biocomposites, which were able to reduce the number of colonies in the above six logs compared to the control culture. The strong antibacterial effect of matrix 3 with phage can be explained by the direct contact of swollen bacterial cells with phage particles immobilized in the chitosan polymer structure, followed by phage propagation and lysis of the bacterial cell. Moreover, there was no significant difference in the PAO1 reduction obtained for matrix 3 without phage, compared to the PAO1 control. However, the matrix based on MKCh without the addition of phages also showed some antibacterial activity by inhibiting the proliferation of PAO1. This is due to the biological activity of chitosan itself, which is known for its antibacterial effect; therefore, no increase in PAO1 was observed.

## Discussion

In this study, the novel chitosan-based matrix was prepared and characterized to serve as a carrier for lytic phages. Literature provides many examples of the positive effect of chitosan biocomposites on the healing process of skin wounds associated with its high biocompatibility, non-toxicity, absorbency, and antibacterial effect (Nesovica et al. 2019). Chitosan dressings are a widely tested group of biomaterials, in the area of which scientists are still discovering new possibilities for innovative solutions. The use of chitosan as a dressing is primarily related to the hemostatic and antimicrobial properties of this polymer. It is also possible to combine it with active substances, with antibiotics, phages, or silver ions, to improve its therapeutic effect on hard-to-heal wounds and burns (Baranwal et al. 2018; Pellá et al. 2018). One of the strategies for designing antimicrobial dressings is the use of polymer matrices, including chitosan, in the supply of phages in the wound environment as a bactericidal agent (Zhang et al. 2020). The possibility of synthesis of chitosan-based carriers of the abovementioned biocidal agents as semi-solid preparations (e.g., in the form of a hydrogel) or solid preparations (e.g., membrane, sponge, nanofibers) allows to “design” their release kinetic from the polymer matrix (Shen et al. 2021).

Numerous studies can be found in the literature, primarily on the methods of phage encapsulation. Phage encapsulation studies have used a variety of hydrophilic and hydrophobic polymers, including agarose, alginate, and chitosan. Phages have been encapsulated in nanospheres and microspheres. In the case of encapsulation, it is important to carefully study and design the triggering factor for phage release; these are primarily solvation, polymer dissolution and erosion, polymer hydrolysis, temperature-induced phase inversion as well as pH and presence of enzymes at the application site. It is therefore a difficult process, requiring the design of an appropriate coating resistant to many factors. The encapsulation technique was used primarily in the oral administration of phage preparations (Mukhopadhyay et al. 2019).

The chitosan-based material synthesized in this study was in the form of a microcrystalline film (MKCh) to carry phages. Free of salt and excess alkali, the MKCh suspension was concentrated to a paste consistency, which was the basic formulation. The polymer matrix was prepared based on MKCh in the form of a paste diluted with water to a concentration of 1.5%. The MKCh form was then combined with tested phages and dried on Teflon plates.

In the case of treatment of skin wounds, the biopolymer matrix can release the killing agent into the wound environment in a controlled manner or immobilize it in its structure (absorption of wound exudate and eradication of the pathogen) as described by Nesovica et al. (2019). The film applied in our study had a positive effect on the ability to swell with moisture, including the effectiveness of absorption of the bacterial solution, consisting of the microcrystalline form of chitosan (MKCh). Of the three different matrices prepared with MKCh, only matrix 3 showed adequate film flexibility due to the optimal composition and content of plasticizer (glycerin) used in a ratio of 0.6 to the polymer content.

Three well-characterized lytic phages specific to *P. aeruginosa* (KTN4 Myovirus giant, KT28 Myovirus, and LUZ19 Podovirus) were used to be immobilized and tested in terms of their antibacterial potential against *P. aeruginosa* PAO1. Assuming that the antibacterial activity of prepared dressing could be related to the physicochemical properties of phages themselves, we selected viruses differing in taxonomy classification, capsid size, and virion morphology, as well as in recognized receptors (saccharide versus proteinous) to evaluate viral particles’ release profile and the potential interference with chitosan matrix. All selected phages were stable in a wide pH range and tolerant to the environment of the chitosan matrix exhibiting a slightly alkaline pH (Olszak et al. 2018).

A well-designed wound dressing impacts the healing at each step. Its antibacterial activity is important to protect against infection, but also good absorption of exudate is an important feature (Koehler et al. 2018; Choi et al. 2020). Considering the potential purpose of the dressing prototype created in this study, we focused on important application aspects. First of all, the ability of the matrix to swell and absorb water content was tested. Our dressing prototype in the form of a chitosan-based film showed high absorptive properties both in itself and after the addition of phages. The level of water absorption by these materials might be correlated with the types of chemical binding of phage virions with chitosan. The KT28 phage had the highest ability to bind with CH_3_ groups of chitosan by hydrogen bonds, so the matrix 3 modified by KT28 Myovirus exhibited lower swelling properties by limited chemical water molecule-binding groups. LUZ19 Podovirus and KTN4 Myovirus giant could bind with chitosan by saccharide rings, and matrix 3 modified by these phages proved higher water absorption properties by hydrogen bonds.

Secondly, the irregularity of the biocomposite surface in the form of a chitosan film was investigated by SEM. The tested films regardless of phage addition indicated their uneven surface which was directly related to the structure of microcrystalline chitosan (MKCh). This is a great advantage in the context of using biocomposite as contact dressings for better adherence to the wound surface. The results of histological studies indicated in the literature prove that the use of chitosan dressings in the form of membranes stimulates the migration of inflammatory cells and determines the cellular organization during the healing process (Miguel et al. 2019). Clinical data prove that chitosan membranes adhere uniformly to the wound surface, inducing cascade coagulation reactions, reducing pain and itching accompanying the healing process, and supporting the scarring process (Kenawy et al. 2019). In addition, the biological activity of chitosan, widely described in the literature, promotes wound healing, mainly due to its hemostatic and antibacterial properties (Mayet et al. 2014; Mukhopadhyay et al. 2019). Chitosan is also able to bind the endotoxin of gram-negative bacteria, i.e., LPS, which may be an additional advantage of the prototype described in this work (Schneier et al. 2020). Bacterial metabolites and degradation products together with LPS may be secreted into an exuding wound (Brothers et al. 2015). Thanks to the absorbent capacity described above and the chitosan present in the composition of the matrix, the healing process can be accelerated. In our study, the addition of phages actively reduces the abundance of *P. aeruginosa*. The antibacterial activity of phages against *P. aeruginosa* was also demonstrated by Mendes et al. (2013) in rodent and pig models. Their results showed that the phages were effective in reducing *Staphylococcus aureus* and *P. aeruginosa* population densities in wound healing. The advantage of the matrix designed in our study is the ability to absorb bacteria along with the exudate into the matrix, where active phages propagate and lyse bacterial cells. A well-developed morphological surface of the matrix and appropriate flexibility promote adhesion and provide a good protective barrier for the wound against an unfavourable external environment (Mi et al. 2003).

The laser interferometry technique combined with the standard colony count methods showed that the number of virions released from the matrix structure was strongly limited. Similar studies conducted by Dorotkiewicz-Jach et al. (2023) confirmed the immobilization of the same phages on a matrix of another polysaccharide—agarose. The lack of significant diffusion of phage particles within the chitosan matrix might be explained by the difference in net charge between the capsid (negatively charged) and the tail (positively charged) causing directional immobility in the positively charged biopolymer matrix as demonstrated by Meyer et al. (2017). This polarization may lead to the orientation of phages within the examined structure, which is confirmed by other studies by Nogueira et al. (2017) available in the literature. We assume that phages regardless of the virion size and affinity to polysaccharide or protein structures were immobilized within a positively charged moieties of chitosan matrix. The ability of chitosan to bind microparticles and macroparticles is primarily due to the presence of two types of reactive functional groups: an amino group and two hydroxyl groups—at the third and sixth carbon atoms in the saccharide ring, as extensively described in the works of Zhang et al. (2020) and Ji et al. (2022). The positively charged chitosan moieties and negatively charged capsids of phages led to the strong adsorption of the phages on the matrix molecules, which was confirmed by the FT-IR analysis. Nevertheless, immobilized phage particles retained their infectivity via unbound point tail fibers that were still able to recognize bacterial surface receptors and infect the cells. This is important from the therapeutic point of view providing lytic phages in the designed chitosan occlusive dressing. The conducted antibacterial assay according to ISO 22196 (2011) showed very high antibacterial activity in direct contact with PAO1 culture for all three tested phages. Studies previously reported in the literature by Danis-Wlodarczyk et al. (2015) confirm the effectiveness of KTN6 and KT28 against this strain. The biopolymer matrix we used did not disturb the antibacterial properties of the phages embedded in the matrix.

**Fig. 6:**
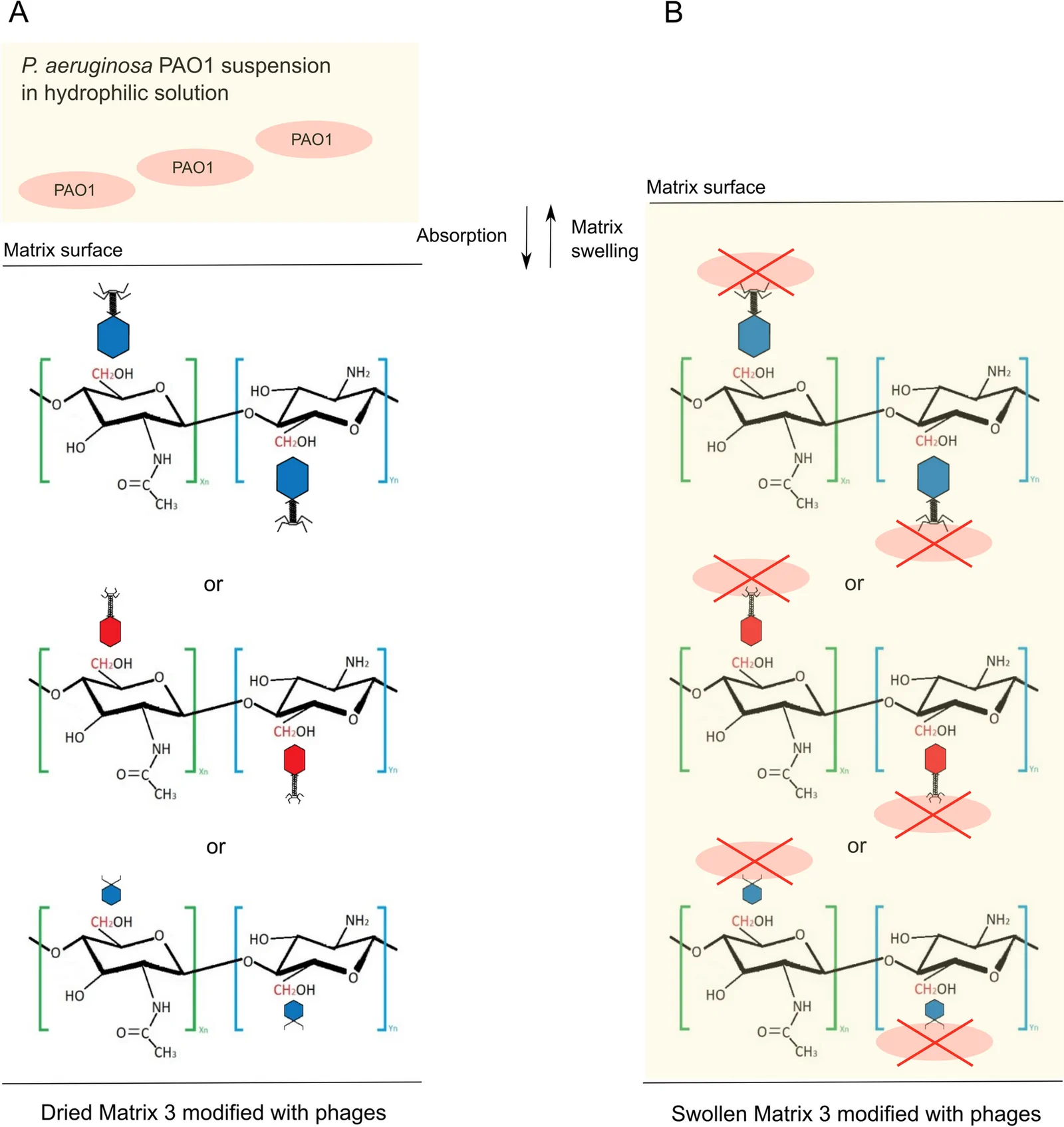
The antibacterial activity concept of matrix 3 modified with phages against *P. aeruginosa* PAO1 cells. **A** “Ready-to-use” dried matrix 3 combined with phages presented the potential interaction of chitosan CH_2_ groups with phage particles; **B** the putative mechanism of antibacterial activity of matrix 3 combined with phages shows both bacterial solution swelling and phage-mediated lysis by viral particles immobilized in the polymer structure

The chitosan film described in this article showed anti-pseudomonal potential. The swelling ability and a positive result in binding phages to the matrix structure allowed them to have an antibacterial effect in direct contact with *P. aeruginosa* PAO1 suspension absorbed by the biocomposite. The concept of antibacterial mode of action for designed material is presented in Fig. 6.

It can be concluded that the prototype of the MKCh membrane dressing with immobilized lytic phages has a high antibacterial potential, and the use of chitosan to create combined dressings is justified by its haemostatic, antibacterial, biocompatible, and biodegradable properties. The preliminary results presented in this paper provide a good basis for conducting more extensive research on the proposed prototype of an occlusive dressing with an active antibacterial effect.

## Data Availability

All data generated or analyzed during this study are included in this published article.
